# Publishing the Transactions of the Georgia Medical Association

**Published:** 1886-04-20

**Authors:** James B. Baird

**Affiliations:** Atlanta


### PUBLISHING THE TRANSACTIONS OF THE GEORGIA MEDICAL ASSOCIATION.

We publish below a communication from Dr. James B. Baird, the former Secretary of the Georgia Medical Association, in regard to the present policy of publishing the Transactions of the Association in a private journal. We asked his opinion because he is probably better acquainted with the cost of publishing its Transactions than any other disinterested party of whom we could inquire. He clearly shows that the plea of poverty, which was *the only one presented by those interested* in the abandonment of the former plan, is without foundation in fact; and this would seem to be sufficient to show to those gentlemen who were thereby induced to favor the scheme that a mistake was made by them, and it ought to be no longer continued.

We cannot believe that the large body of those who voted for this action understood its bearings, or realized the inferences clearly deducible from such action. One of them is very forcibly pointed out by Dr. Baird when he remarks that the Association virtually says to a member that he shall subscribe and pay for a particular journal or he can no longer remain a member of the Association—thus prescribing a condition of membership heretofore unknown, and certainly not authorized by the constitution of the Association. Another inference is, that it bestows upon a private journal the patronage and influence of the Association, and unjustly makes the impression abroad that it is the principal organ of the Profession in the State, and the only one worthy of the confidence and patronage of the profession. This inference is sustained in the fact that, while many journals seek and gladly pay for such original matter, this society voluntarily furnishes the choice papers of its members to a private journal; not only so, but pays for the privilege of doing so a sum twice as much as would suffice to put the papers in accessable and permanent form.

And now we would respectfully ask, Did the members of the Association knowingly and intentionally lend their aid and influence toward the furtherance of such objects, and impressions as we have named? We do not believe that they did; and yet such inference is deducible from their action, and we think, now that the matter is fairly before them, that they cannot fail to see that injustice has been done, not only to competing journals equally worthy, and their subscribers, but to competing medical institutions not represented by the favored journal—to the members of the Association at large, and especially to the integrity of those who unwittingly sustained this action.

It is possible that there are some who will attribute these remarks to personal interest, as from editors of a competing journal, but we have endeavored to discuss the subject in a spirit of fairness and candor, and with an honest purpose to vindicate the truth and to uphold the dignity and honor of the Profession—objects which, in times past, no one can say that our journal has ever failed to advocate and promote. But read the following from Dr. Baird :

*Editors Southern Medical Journal:*

GENTLEMEN—I am in receipt of your favor of the 8th instant, in which you request my opinion, as former secretary of the Medical Association of Georgia, concerning the action of the Association, at its meeting in Savannah last year, providing for a change in the method of publishing the Transactions of that body, and directing that the papers presented at the annual sessions and the record of proceedings should be turned over to a medical journal for publication. You ask my views as to the financial necessity for this procedure, and if, in my judgment, "the Association had the constitutional right to take such action ; and if it did, was it wise in policy, correct in principle, or just to the members of the Association ?"

My experience as Secretary of the Association from 1877 to 1881, and as a member of the Committee on Publication continuously from 1872 to 1885, enables me to speak with considerable confidence upon the subject of your inquiry, and I cheerfully respond, as succinctly and as clearly as I can, to your polite request. I do so the more readily as I may not be able to attend the meeting next month, and desire to be on record in opposition to this unfortunate innovation.

Providential causes have prevented my presence at the last two meetings of the Association, and, consequently, I am ignorant of the arguments which were advanced in favor of the radical change in the customary mode of issuing our Annual Transactions ; but that fact need not prohibit a candid review of the situation, or an honest and dispassionate expression as to the expediency of the step.

Let us inquire into the question of economy.

Four hundred copies of Transactions, of 163 pages each, were issued in 1876 at a total cost of $276.25, delivered, or 55½ cents per copy.

Six hundred copies, of 200 pages each, were issued in 1877 at a net cost—after deducting the amount received for a few approved advertisements—of $182, or 30⅛ cents each.

Four hundred copies, of 280 pages each, were issued in 1878 at a net cost of 60¼ cents per copy.

Four hundred copies, of 288 pages each, were issued in 1879 at a net cost of 49⅓ cents per copy.

Four hundred copies, of 252 pages each, were issued in 1880 at a net cost of 66¾ cents per copy—the highest price paid for the Transactions during the five years that I was in the Secretary's chair.

By referring to the elaborate report of my immediate successor, Dr. A. Sibley Campbell, it will be perceived that the net cost to the Association of the four hundred copies issued under his administration, in the very handsome cloth covers which he devised, was only 59¾ cents each. The gross cost of this elegant edition, including wrappers and postage, without any credit or deduction whatever, was only $1.22 1-5 per copy, of 318 pages each.

The gross cost of the edition of 1883, issued in the same attractive and expensive style, was $1.08½ per copy, of 275 pages. The net cost to the Association was about 53½ cents.

If I am correctly informed, the Transactions for 1885, under the new system, have cost every member of the Association, who has received them, $2 !

The roll of 1884 contains 264 names. Two dollars each from 91 members would cover the net cost of the edition of 600 copies in 1877. This would allow a copy for every member, and leave a surplus of 336 copies for general distribution, if so desired. I believe, however, that the dues are now $3 per annum. Hence 60 prompt paying members would insure the publication of a respectable volume ; and surely the incidental expenses—which ought not to be large—could be procured from the remaining 204 members.

According to the published reports of the officers, the total receipts from all sources, for 1879, amounted to $530.09. The receipts for 1880 are not stated, but some $400 were expended. The presumption is that more than this amount was received. In 1881, a total of $558.77 was collected ; in 1882, $436.20, and in 1883, $458.14.

Contemplation of these facts, hastily grouped, will, I think, effectually dispose of the poverty plea.

The constitution contains no express provision regulating the manner of publishing the Transactions. It seems to have been understood that the volume would be printed, and the arrangement of the details have been left to the committee on publication. It is, however, the law of the organization that failure to pay dues for two successive years shall forfeit membership in the Association. In its last analysis, then, we find this anomalous state of things : If you do not subscribe for a particular medical journal—which is a private enterprise, and with which the Association is in no wise concerned, and over which it has no control—you will be expelled from membership from your State Society !

There may be reasons—good and sufficient reasons to the individual—why he does not wish to receive the journal selected by the majority voting. Now, ought the Association to say to such a member, " You shall pay for this journal, or you cannot retain your connection with this organization ? " Has the Association the right to prescribe this act as a condition of membership—which is the practical effect of this arrangement—or to use its moral and pecuniary power to build up an enterprise which is entirely foreign to it, and independent of it ? I think not. The plan is, in my judgment, a grave mistake, which will not promote harmony at home, or increase our influence abroad.

I hope to see the Association promptly forsake this new departure, and speedily return to the good old way, so that the History of the organization may be preserved, and its record safely kept. If the voluntary papers and reports of sections cannot, for the lack of funds, be printed with the record of the business proceedings, by the Association, let all such contributions be returned by the publishing committee to the writers, as is already provided by resolution of the body, and as was done in 1875, to be published as their respective authors may see proper.

I am aware that, in some quarters, it is considered enlightened and progressive to decry "musty volumes of Transactions," and to bemoan the burial of valuable papers in these sepulchers of ancient form. The limited audience is lamented by the aspiring author, and he grieves that the fruit of his genius should be hid under a bushel. We need only remind these ambitious writers, who are anxious to proclaim their special skill, who sedulously count the profits of their productions when widely read, and who inveigh thus against the time-honored custom of issuing an annual volume, that their contributions are entirely voluntary, and that it is not to the general interest of the medical profession to loosen the bands, to lessen the restraints, or to neglect the discipline which thorough organization is supposed to secure.

How far the modern enterprising methods, more or less in vogue among medical men, go toward exalting the standing, maintaining the dignity, increasing the influence, and augmenting the usefulness of the profession, let the veterans in the worthy cause declare. For myself, Messrs. Editors, I am coustrained to say that, in my opinion, the action of the Association under discussion was unnecessary, unwise, and unjust

JAMES B. BAIRD.

ATLANTA, March 16, 1886.

